# The Association Between Video Gaming and Psychological Functioning

**DOI:** 10.3389/fpsyg.2019.01731

**Published:** 2019-07-26

**Authors:** Juliane M. von der Heiden, Beate Braun, Kai W. Müller, Boris Egloff

**Affiliations:** ^1^Department of Psychology, Johannes Gutenberg University Mainz, Mainz, Germany; ^2^Department of Psychosomatic Medicine, University Medical Center, Mainz, Germany

**Keywords:** computer games, video gaming behavior, game genres, coping, psychological health

## Abstract

Video gaming is an extremely popular leisure-time activity with more than two billion users worldwide (Newzoo, 2017). However, the media as well as professionals have underscored the potential dangers of excessive video gaming. With the present research, we aimed to shed light on the relation between video gaming and gamers’ psychological functioning. Questionnaires on personality and psychological health as well as video gaming habits were administered to 2,734 individuals (2,377 male, 357 female, *M*_age_ = 23.06, *SD*_age_ = 5.91). Results revealed a medium-sized negative correlation between problematic video gaming and psychological functioning with regard to psychological symptoms, affectivity, coping, and self-esteem. Moreover, gamers’ reasons for playing and their preferred game genres were differentially related to psychological functioning with the most notable findings for distraction-motivated players as well as action game players. Future studies are needed to examine whether these psychological health risks reflect the causes or consequences of video gaming.

## Introduction

Video gaming is a very popular leisure activity among adults (Pew Research Center, 2018). The amount of time spent playing video games has increased steadily, from 5.1 h/week in 2011 to 6.5 h/week in 2017 (The Nielsen Company, 2017). Video gaming is known to have some benefits such as improving focus, multitasking, and working memory, but it may also come with costs when it is used heavily. By spending a predominant part of the day gaming, excessive video gamers are at risk of showing lower educational and career attainment, problems with peers, and lower social skills (Mihara and Higuchi, 2017). On the one hand, video game use is widespread, and it may come with certain precursors as well as consequences. On the other hand, little is known about the relations between various video gaming habits and psychological functioning. This study aims to shed light on these important relations using a large sample.

A video game is defined as “a game which we play thanks to an audiovisual apparatus and which can be based on a story” (Esposito, 2005). In the last few years, the amount of scientific research devoted to video game playing has increased (e.g., Ferguson, 2015; Calvert et al., 2017; Hamari and Keronen, 2017). Most scientific studies in this area of research have focused on the extent of video game play and its diverse correlates. While some researchers have emphasized the benefits of game playing and even suggested a therapeutic use of video games (Primack et al., 2012; Granic et al., 2014; Colder Carras et al., 2018), others have been intrigued by its potential dangers (Anderson et al., 2010; Müller and Wölfling, 2017).

Parents and professionals may be worried about their excessively playing children being “addicted.” However, problematic and potentially addictive video game use goes beyond the extent of playing (in hours per week; Skoric et al., 2009). It also includes such issues as craving, loss of control, and negative consequences of excessive gaming. While it is still a matter of debate whether problematic video game play should be considered a *behavioral addiction*, its status as a mental disorder has been clarified since the release of the DSM-5 in 2013. In the DSM-5, the American Psychiatric Association (2013) defined *Internet Gaming Disorder* with diagnostic criteria closely related to Gambling Disorder. Generally, this decision has been supported by many researchers (e.g., Petry et al., 2014) but has also caused controversies. Researchers have criticized the selection of diagnostic criteria and the vague definition of the *Internet Gaming Disorder* construct, which excludes offline games from being related to addictive use (e.g., Griffiths et al., 2016; Bean et al., 2017).

Several studies, literature reviews, and meta-analyses have focused on the correlates of problematic video gaming, usually assessed as a continuum with addiction marking the upper end of the scale (e.g., Ferguson et al., 2011; Kuss and Griffiths, 2012). The degree of addictive video game use has been found to be related to personality traits such as low self-esteem (Ko et al., 2005) and low self-efficacy (Jeong and Kim, 2011), anxiety, and aggression (Mehroof and Griffiths, 2010), and even to clinical symptoms of depression and anxiety disorders (Wang et al., 2018). Potential consequences of video game use have been identified as well, such as a lack of real-life friends (Kowert et al., 2014a), stress and maladaptive coping (Milani et al., 2018), lower psychosocial well-being and loneliness (Lemmens et al., 2011), psychosomatic problems (Müller et al., 2015; Milani et al., 2018), and decreased academic achievement (Chiu et al., 2004; Gentile, 2009). Effect sizes have varied widely across studies (Ferguson et al., 2011). There seem to be sex and age differences with regard to video gaming behavior: potentially problematic video gaming was found to be more likely among males than females (e.g., Greenberg et al., 2010; Estévez et al., 2017), and among younger gamers (Rehbein et al., 2016).

In addition to looking at problematic video game use and its relation to psychological functioning, it is relevant to also focus on *why* individuals play video games. Players use video games for very different reasons (Ryan et al., 2006; Yee, 2006) such as to distract themselves from daily hassles or because they enjoy the social relationships they have developed in the virtual world. Potentially problematic video gaming has been found to be related to various reasons for playing such as coping and escape (Hussain and Griffiths, 2009; Schneider et al., 2018), socialization (Laconi et al., 2017), and personal satisfaction (Ng and Wiemer-Hastings, 2005). Coping (Laconi et al., 2017), social interaction, and competition were among the main reasons for gaming among males but not among females (Lucas and Sherry, 2004). Mixed results emerged concerning age differences (Greenberg et al., 2010), but especially younger gamers seemed to be motivated for video gaming by social interactions (Hilgard et al., 2013). However, so far it remains unclear to what extent people’s various reasons for playing video games are differentially related to their psychological functioning.

Besides investigating the links between potentially problematic video game use and psychological functioning as well as between reasons for playing video games and psychological functioning, it is relevant to also look at which *game genres* individuals prefer. Correlates of preferences for certain game genres (e.g., simulation, strategy, action, role-playing) are cognitive enhancement (Dobrowolski et al., 2015; Bediou et al., 2018), but also the amount of time spent playing (Lemmens and Hendriks, 2016; Rehbein et al., 2016) and psychopathological symptoms (Laconi et al., 2017). Males were shown to prefer action and strategy games, whereas females showed a preference for games of skill (Scharkow et al., 2015; Rehbein et al., 2016). Younger gamers seemed to prefer action games, older players more so games of skill (Scharkow et al., 2015). However, it is not yet understood to what extent preferences for certain video game genres are differentially related to psychological functioning.

Typically, research has focused merely on violent video games (e.g., Anderson and Bushman, 2001; Elson and Ferguson, 2014) or one specific game within one specific game genre (frequently World of Warcraft; Graham and Gosling, 2013; Visser et al., 2013; Herodotou et al., 2014), thereby neglecting the variety of possible gaming habits across various game genres.

In the present study, our objective was to examine the relation between video gaming and psychological functioning in a fine-grained manner. For this purpose, we examined psychological functioning by employing various variables such as psychological symptoms, coping strategies, and social support. Likewise, we assessed video gaming in a similarly detailed way, ranging from (a) problematic video game use, (b) the reasons for playing, to (c) the preferred game genres. This strategy prevented us from making potentially invalid generalizations about video gaming in general and allowed us to examine the spectrum of gaming habits and the respective relations between such habits and a diverse set of variables representing psychological functioning.

Playing video games excessively should be appealing to individuals with poor psychological functioning because games allow people to avoid their everyday problems and instead immerse themselves in another environment (Taquet et al., 2017). Moreover, video games offer people a chance to connect with other people socially despite any more or less evident psychological problems they may have (Kowert et al., 2014b; Mazurek et al., 2015). On the other hand, potentially problematic video game use may also lead to psychological problems because it reduces the amount of time and the number of opportunities gamers have to practice real-life behavior (Gentile, 2009). Thus, we expected to find a negative correlation between problematic video gaming and variables representing psychological functioning such that we expected more potentially problematic video game use to be related to dysfunctional coping strategies (Wood and Griffith, 2007), negative affectivity (Mathiak et al., 2011), and poor school performance (Mihara and Higuchi, 2017). Moreover, we expected to find differential correlates of people’s reasons for playing video games and their psychological functioning: Playing for escape-oriented reasons such as distraction should go along with diverse indices of poor psychological functioning (Király et al., 2015), whereas playing for gain-oriented reasons such as the storyline or the social connections in the game should be related to adequate psychological functioning (Longman et al., 2009). Also, we expected to find people’s preferred game genres (e.g., strategy, action) to be differentially related to their psychological functioning (Park et al., 2016). Finally, we aimed to shed light on the unique contribution of each measure of psychological functioning to the prediction of problematic video game use.

## Materials and Methods

### Participants^1^

A total of *N* = 2,891 individuals (2,421 male, 470 female) with a mean age of 23.17 years (*SD* = 5.99, Range: 13–65) participated in our study. Of these participants, *N* = 2,734 (95%) confirmed their use of video games and were thus included in further analyses (2,377 male, 357 female, with a mean age of 23.06 years; *SD* = 5.91, Range: 13–65). The distribution of participants with regard to sex and age mirrors the findings of past research with males and younger individuals being more likely to play video games (e.g., Griffiths et al., 2004). Participants’ place of residence was Germany.

### Procedure and Instruments^2^

We posted links to our online questionnaire on various online forums as well as on popular online game sites. To achieve heterogeneity of the sample, no exclusion criteria other than having access to the Internet and understanding German were specified. As an incentive to participate in the study, four vouchers of 50€ were raffled.

#### Video Gaming

##### Potentially problematic video game use

The AICA-S, the Scale for the Assessment of Internet and Computer game Addiction (Wölfling et al., 2016), was used to assess participants’ gaming behavior with regard to potential problematic use. Based on the DSM criteria for Internet Gaming Disorder (tolerance, craving, loss of control, emotion regulation, withdrawal, and unsuccessful attempts to cut back), this standardized self-report scale consists of 15 items usually with a five-point scale ranging from 1 (*never*) to 5 (*very often*). The final score (Min = 0, Max = 27 points) is computed using weighted scoring (items with an item-total correlation > 0.55 in the norm sample are weighted double; Wölfling et al., 2011). The AICA-S score can be used to differentiate between regular (0–6.5 points) and problematic use of video games (7–13 points: abuse; 13.5–27 points: addiction). In our sample, *N* = 2,265 (83%) were identified as regular gamers, and *N* = 469 (17%) as problematic gamers. We used the AICA-S as a continuous variable for all further analyses (*M* = 3.98, *SD* = 3.22, Range: 0–24). The instrument has been validated for different age groups in the general population and in clinical samples (Müller et al., 2014a, 2019, but note small sample size; Müller et al., 2014b). Cronbach’s alpha was α = 0.70. As expected, the AICA-S score was correlated with male sex (*r* = 0.17^∗∗∗^) and age (*r* = –0.15^∗∗∗^). On average, participants played video games for *M* = 4.09 hours per weekday (*SD* = 4.44, Range: 0–24), and *M* = 4.21 h per day at the weekend (*SD* = 2.99, Range: 0–24).

##### Reasons for playing

Gamers indicated how often they played video games for certain reasons. They rated each of 10 reasons separately on Likert scales ranging from 1 (*never*) to 4 (*very often*). The most prevalent reasons were relaxation (*M* = 2.96, *SD* = 0.91), amusement (*M* = 2.94, *SD* = 0.85), and because of the storyline (*M* = 2.67, *SD* = 1.10).

##### Game genres

Gamers were asked how often they usually played various video game subgenres such as first-person shooter, round-based strategy, massively multiplayer online role-playing games (MMORPGs), life simulations, and others. Ratings were made on Likert scales ranging from 1 (*never*) to 4 (*very often*). Using Apperley’s (2006) classification of game genres, we categorized the subgenres into the main genres action (*M* = 2.54, *SD* = 0.84), strategy (*M* = 2.13, *SD* = 0.80), role-playing (*M* = 2.01, *SD* = 0.73), and simulation (*M* = 1.58, *SD* = 0.44). A cluster for unclassified subgenres (*M* = 1.54, *SD* = 0.39) was added to additionally account for such subgenres as jump’n’runs and games of skill. Descriptive statistics and intercorrelations for all measures (including sex and age) are presented in Supplementary Tables S1–S4.

#### Psychological Functioning

Participants provided ratings of their psychological functioning on the following constructs:

##### General psychopathology

The SCL-K-9 (Klaghofer and Brähler, 2001), a short version of the SCL-90-R (Derogatis, 1975), was administered to assess participants’ subjective impairment regarding psychological symptoms (somatization, obsessive-compulsive, interpersonal sensitivity, depression, anxiety, hostility, phobic anxiety, paranoid ideation, and psychoticism). The SCL-K-9 score is strongly correlated with the original score of the SCL-90-R (*r* = 0.93). The 9 items were answered on 5-point Likert-type scales ranging from 1 (*do not agree at all*) to 5 (*agree completely*). Cronbach’s alpha was satisfactory (α = 0.77).

##### Coping

We assessed 10 coping strategies with the Brief COPE (Carver, 1997; German version by Knoll et al., 2005), which is the shorter version of the COPE (Carver et al., 1989): self-distraction, denial, substance use, venting, self-blame, behavioral disengagement, acceptance, active coping, planning, and positive reframing. The two items per subscale were administered on 5-point Likert-type scales ranging from 1 (*never*) to 5 (*very often*). Intercorrelations of the two items per subscale ranged from *r* = 0.32, *p* < 0.001 for positive reframing to *r* = 0.78, *p* < 0.001 for substance use (with one exception: *r* = -0.05, *p* = 0.01 for self-distraction).

##### Affect

We measured *general affect* as a trait and *affect during video gaming* as a state using the German version (Krohne et al., 1996) of the Positive and Negative Affect Schedule (PANAS; Watson et al., 1988). On a 5-point Likert-type scale ranging from 1 (*not at all*) to 5 (*completely*), participants rated the intensity of 20 adjectives. Cronbach’s alpha was α = 0.78 for general positive affect, α = 0.83 for general negative affect, α = 0.85 for positive affect while playing, and α = 0.83 for negative affect while playing.

##### Shyness

The measure for the assessment of shyness in adults (Asendorpf, 1997) consists of 5 items that were answered on a 5-point Likert-type scale ranging from 1 (*not at all*) to 5 (*completely*). Cronbach’s alpha was excellent (α = 0.86).

##### Loneliness

We administered the German version (Elbing, 1991) of the NYU Loneliness Scale (Rubenstein and Shaver, 1982). The 4 items were answered on 5- to 6-point Likert-type scales. Cronbach’s alpha was satisfactory (α = 0.79).

##### Preference for solitude

A 10-item measure of preference for solitude (Nestler et al., 2011) was answered on a 6-point Likert-type scale ranging from 1 (*not at all*) to 6 (*completely*). Cronbach’s alpha was excellent (α = 0.86).

##### Life satisfaction

Participants answered a one-item life satisfaction measure on a 4-point Likert-type scale ranging from 1 (*not at all*) to 4 (*completely*).

##### Self-esteem

We administered the German version (von Collani and Herzberg, 2003) of the Rosenberg Self-Esteem Scale (RSES; Rosenberg, 1979). The 10 items were answered on a 4-point Likert-type scale ranging from 1 (*not at all*) to 4 (*completely*). Cronbach’s alpha was excellent (α = 0.88).

##### Self-efficacy

We administered a 10-item generalized self-efficacy scale (Schwarzer and Jerusalem, 1995), which was answered on a 4-point Likert-type scale ranging from 1 (*not at all*) to 4 (*completely*). Cronbach’s alpha was excellent (α = 0.86).

##### Social support and friends

We administered the perceived available social support subscale from the Berlin Social Support Scales (BSSS; Schwarzer and Schulz, 2003). The 8 items were answered on a 5-point Likert-type scale ranging from 1 (*not at all*) to 5 (*completely*). Cronbach’s alpha was excellent (α = 0.94). Participants indicated how many offline friends and offline acquaintances they had (*r* = 0.44, *p* < 0.001) as well as how many online friends and online acquaintances they had (*r* = 0.33, *p* < 0.001). Due to left-skewed distributions, we logarithmized the data before aggregation.

##### Grades

Participants reported their grade point average. German grades are assessed on a scale that ranges from 1 (*excellent*) to 6 (*insufficient*). Thus, higher scores indicate worse grades.

Participants further reported their sex and age. Both were used as control variables in further analyses.

### Analyses

In a first step, we computed zero-order correlations between the video gaming variables and the measures of psychological functioning. In a second step, we computed partial correlations in which we controlled for sex and age because past research has repeatedly shown that sex and age are correlated with both video gaming (Homer et al., 2012; Mihara and Higuchi, 2017) and psychological functioning (Kessler et al., 2007; Nolen-Hoeksema, 2012). Finally, we explored the unique contribution of each measure of psychological functioning to the prediction of potentially problematic video gaming. Therefore, we computed regressions with potentially problematic video gaming as the dependent variable and sex, age, and the measures of psychological functioning as predictors (entered simultaneously into the regression equation). By employing this procedure, we were able to determine the effect that each variable had over and above the other ones. For instance, we could identify whether general psychopathology was predictive of potentially problematic video game use when the influence of all other variables (e.g., shyness, loneliness, and others) was held constant.

Additionally, we included analyses regarding sex and age differences in the link between video gaming and psychological functioning. Since we collected a self-selected sample where different sexes and age groups were not represented equally, our findings are only preliminary, but may stimulate future research.

## Results

### Potentially Problematic Video Game Use and Psychological Functioning

First, we examined whether potentially problematic video game use was related to various psychological functioning variables. As can be seen in Table 1, the results for the zero-order correlations were similar to those for the partial correlations in which we controlled for sex and age. A medium-sized positive relation to the potentially problematic use of video games emerged for the presence of psychological symptoms including depression, anxiety, and hostility. Furthermore, several coping strategies were differentially associated with the potentially problematic use of video games: Self-blame and behavioral disengagement showed the strongest positive relations to potentially problematic video game use, followed by denial, acceptance, substance use, self-distraction, and venting. Planning, active coping, and, to a lesser extent, positive reframing were negatively associated with the potentially problematic use of video games. Moreover, the association with potentially problematic video game use was negative for general positive affect and positive and larger in size for general negative affect. However, potentially problematic video game use was clearly positively associated with the experience of both positive and negative affect while playing. Further, a preference for solitude, shyness, and loneliness were positively correlated with the potentially problematic use of video games. Lower self-esteem, lower life satisfaction, and, to a lesser extent, poorer perceived social support and lower self-efficacy went along with potentially problematic video game use. There was an association between fewer offline friends and acquaintances but more online connections with potentially problematic video gaming. Finally, poorer performance in school (i.e., higher grades) was related to the potentially problematic use of video games. These results suggest that potentially problematic video gaming goes along with poor psychological functioning and vice versa.

**TABLE 1:** Associations between potentially problematic video gaming and psychological functioning.

	**Potentially problematic video gaming**	
	**r**	***r*_part_**
General psychopathology	0.28^∗∗∗^	0.31^∗∗∗^
**Coping**		
Self-distraction	0.13^∗∗∗^	0.14^∗∗∗^
Denial	0.17^∗∗∗^	0.16^∗∗∗^
Substance use	0.15^∗∗∗^	0.15^∗∗∗^
Venting	0.09^∗∗∗^	0.14^∗∗∗^
Self-blame	0.23^∗∗∗^	0.24^∗∗∗^
Behavioral disengagement	0.24^∗∗∗^	0.24^∗∗∗^
Acceptance	0.17^∗∗∗^	0.16^∗∗∗^
Active coping	–0.13^∗∗∗^	–0.11^∗∗∗^
Planning	–0.14^∗∗∗^	–0.11^∗∗∗^
Positive reframing	–0.06^∗∗^	–0.05^∗∗^
**Affect**		
Positive affect in general	–0.15^∗∗∗^	–0.16^∗∗∗^
Negative affect in general	0.22^∗∗∗^	0.23^∗∗∗^
Positive affect while playing	0.24^∗∗∗^	0.21^∗∗∗^
Negative affect while playing	0.29^∗∗∗^	0.26^∗∗∗^
Shyness	0.20^∗∗∗^	0.21^∗∗∗^
Loneliness	0.16^∗∗∗^	0.16^∗∗∗^
Preference for solitude	0.18^∗∗∗^	0.22^∗∗∗^
Life satisfaction	–0.20^∗∗∗^	–0.21^∗∗∗^
Self-esteem	–0.27^∗∗∗^	–0.28^∗∗∗^
Self-efficacy	–0.16^∗∗∗^	–0.17^∗∗∗^
Social support	–0.20^∗∗∗^	–0.18^∗∗∗^
Friends and acquaintances offline	–0.09^∗∗∗^	–0.13^∗∗∗^
Friends and acquaintances online	0.21^∗∗∗^	0.20^∗∗∗^
Grade point average	0.24^∗∗∗^	0.22^∗∗∗^

*Ns ranged from 2,732 to 2,734. Potentially problematic video gaming was assessed with the AICA-S. Grade point average: higher values reflect poorer school performance. Zero-order correlation coefficients (r) and partial correlation coefficients (r_*part*_, controlled for sex and age) are presented. ^∗∗^p < 0.01 and ^∗∗∗^p < 0.001.*

### Reasons for Playing Video Games and Psychological Functioning

Second, we investigated whether players’ reasons for playing video games were differentially related to the psychological functioning variables. Table 2 presents the partial correlations, controlling for sex and age. Using video games to *distract oneself* from stress was clearly connected to a high level of psychological symptoms. Distraction-motivated gamers preferred coping strategies such as self-blame, behavioral disengagement, self-distraction, denial, substance use, venting, and acceptance, but they neglected active coping and planning. They showed less general positive affect and more negative affect both in general and while playing as well as more positive affect while playing. These gamers further reported low self-esteem and low life satisfaction, loneliness, a preference for solitude, shyness, a lack of self-efficacy and social support, and poor achievement in school. A similar but somewhat less extreme picture was revealed for gamers who played video games in order to *have something to talk about*. However, these gamers reported more online connections. Gamers who played video games to *improve their real-life abilities* also reported more online connections. In addition, these gamers showed higher levels of general positive affect. The strongest association with online friends and acquaintances emerged, as expected, for gamers who played *because of the social relations* in the virtual world. Although all reasons for playing video games were related to positive affect while playing, the strongest associations emerged for gamers who played *because of the social relations*, to *stimulate their imagination*, and for *curiosity*. It is interesting that, for gamers who played video games *because of the storyline* and for *relaxation*, there was a relation only to positive but not to negative affect while playing. Reasons for playing were only weakly related to sex and age (see Supplementary Table S2). In sum, several reasons for playing video games were differentially associated with psychological functioning.

**TABLE 2:** Associations between reasons for playing video games and psychological functioning.

	**Reasons for playing**									
	***r*_part_**									
	**AMUSE**	**DISTR**	**STORY**	**RELAX**	**IMAGI**	**REAL**	**SOCIAL**	**TALK**	**AVATA**	**CURIO**
General psychopathology	0.07^∗∗^	0.26^∗∗∗^	0.04^+^	0.01	0.09^∗∗∗^	0.08^∗∗∗^	0.06^∗∗^	0.13^∗∗∗^	0.09^∗∗∗^	0.09^∗∗∗^
**Coping**										
Self-distraction	0.08^∗∗∗^	0.15^∗∗∗^	0.09^∗∗∗^	0.07^∗∗∗^	0.19^∗∗∗^	0.09^∗∗∗^	–0.01	0.11^∗∗∗^	0.05^*^	0.13^∗∗∗^
Denial	0.04^*^	0.15^∗∗∗^	–0.02	0.01	0.08^∗∗∗^	0.13^∗∗∗^	0.07^∗∗∗^	0.18^∗∗∗^	0.09^∗∗∗^	0.04^*^
Substance use	0.04^+^	0.13^∗∗∗^	–0.01	–0.01	–0.03	0.01	0.02	0.04^*^	0.08^∗∗∗^	0.04^+^
Venting	0.04^*^	0.12^∗∗∗^	0.03	0.02	0.06^∗∗^	0.08^∗∗∗^	0.01	0.10^∗∗∗^	0.08^∗∗∗^	0.06^∗∗^
Self-blame	0.07^∗∗∗^	0.18^∗∗∗^	0.02	0.02	0.04^+^	0.00	0.03	0.06^∗∗^	0.06^∗∗^	0.06^∗∗^
Behavioral disengagement	0.08^∗∗∗^	0.16^∗∗∗^	0.03^+^	–0.02	0.05^∗∗^	0.05^*^	0.02	0.12^∗∗∗^	0.08^∗∗∗^	0.07^∗∗∗^
Acceptance	0.09^∗∗∗^	0.10^∗∗∗^	0.03	0.07^∗∗∗^	0.02	0.04^+^	0.02	0.07^∗∗∗^	0.05^∗∗^	0.06^∗∗^
Active coping	−0.04^*^	–0.10^∗∗∗^	0.06^∗∗^	0.06^∗∗^	0.07^∗∗∗^	0.07^∗∗∗^	0.03	–0.00	0.02	0.08^∗∗∗^
Planning	–0.05^∗∗^	–0.09^∗∗∗^	0.06^∗∗^	0.05^∗∗^	0.06^∗∗^	0.06^∗∗^	0.05^*^	0.00	0.02	0.09^∗∗∗^
Positive reframing	−0.04^*^	–0.05^∗∗^	0.06^∗∗^	0.07^∗∗∗^	0.11^∗∗∗^	0.11^∗∗∗^	0.06^∗∗^	0.06^∗∗^	0.04^*^	0.13^∗∗∗^
**Affect**										
Positive affect in general	–0.06^∗∗^	–0.10^∗∗∗^	–0.01	0.05^*^	0.05^*^	0.10^∗∗∗^	0.08^∗∗∗^	0.00	–0.00	0.07^∗∗∗^
Negative affect in general	0.04^+^	0.20^∗∗∗^	0.05^*^	0.01	0.08^∗∗∗^	0.06^∗∗^	–0.01	0.08^∗∗∗^	0.09^∗∗∗^	0.09^∗∗∗^
Positive affect while playing	0.07^∗∗^	0.09^∗∗∗^	0.13^∗∗∗^	0.19^∗∗∗^	0.21^∗∗∗^	0.19^∗∗∗^	0.20^∗∗∗^	0.15^∗∗∗^	0.19^∗∗∗^	0.20^∗∗∗^
Negative affect while playing	0.07^∗∗^	0.16^∗∗∗^	0.00	0.00	0.05^∗∗^	0.10^∗∗^	0.07^∗∗∗^	0.15^∗∗∗^	0.13^∗∗∗^	0.13^∗∗∗^
Shyness	0.09^∗∗∗^	0.15^∗∗∗^	0.10^∗∗∗^	0.02	0.08^∗∗∗^	0.03	–0.03	0.06^∗∗^	0.05^∗∗^	0.05^∗∗^
Loneliness	0.07^∗∗∗^	0.18^∗∗∗^	0.00	–0.06^∗∗^	0.02	–0.01	0.02	0.09^∗∗∗^	0.01	0.04^+^
Preference for solitude	0.07^∗∗^	0.16^∗∗∗^	0.13^∗∗∗^	0.15^∗∗∗^	0.14^∗∗∗^	0.07^∗∗∗^	–0.03	0.04^*^	0.10^∗∗∗^	0.10^∗∗∗^
Life satisfaction	−0.04^*^	–0.19^∗∗∗^	–0.01	0.04^*^	–0.02	–0.00	0.01	−0.04^*^	–0.00	–0.01
Self-esteem	−0.04^+^	–0.21^∗∗∗^	−0.05^*^	0.02	–0.05^∗∗^	0.00	0.00	–0.09^∗∗∗^	–0.07^∗∗^	−0.04^*^
Self-efficacy	–0.03	–0.15^∗∗∗^	−0.05^*^	0.04^*^	–0.00	0.05^∗∗^	0.09^∗∗∗^	–0.08^∗∗∗^	–0.02	0.00
Social Support	−0.05^*^	–0.12^∗∗∗^	–0.01	0.05^∗∗^	0.00	–0.01	0.01	−0.04^*^	−0.04^+^	–0.03
Friends and acquaintances offline	–0.07^∗∗∗^	–0.07^∗∗∗^	–0.12^∗∗∗^	−0.04^*^	–0.09^∗∗∗^	0.01	0.09^∗∗∗^	0.01	−0.04^*^	–0.08^∗∗∗^
Friends and acquaintances online	0.03	0.03^+^	–0.06^∗∗^	0.02	0.04^+^	0.14^∗∗∗^	0.42^∗∗∗^	0.12^∗∗∗^	0.06^∗∗^	–0.02
Grade point average	0.06^∗∗^	0.12^∗∗∗^	0.03	0.03	0.04^*^	0.03	0.07^∗∗∗^	0.09^∗∗∗^	0.10^∗∗∗^	0.07^∗∗^

Ns ranged from 2,712 to 2,727. Reasons for playing were assessed on a Likert scale ranging from 1 (never) to 4 (very often) and were coded as follows: AMUSE, amusement; DISTR, distraction; STORY, storyline; RELAX, relaxation; IMAGI, stimulation of imagination; REAL, improvement of real-life abilities; SOCIAL, social relationships in the virtual world; TALK, have something to talk about; AVATA, improving the abilities of the avatar; CURIO, curiosity. Grade point average: higher values reflect poorer school performance. Partial correlations (r_*part*_, controlled for sex and age) are presented. Partial correlations (controlled for sex and age) with the AICA-S: r_*part*_ = 0.21^∗∗∗^ for AMUSE, r_*part*_ = 0.32^∗∗∗^ for DISTR, r_*part*_ = 0.02 for STORY, r_*part*_ = 0.13^∗∗∗^ for RELAX, r_*part*_ = 0.12^∗∗∗^ for IMAGI, r_*part*_ = 0.16^∗∗∗^ for REAL, r_*part*_ = 0.29^∗∗∗^ for SOCIAL, r_*part*_ = 0.21^∗∗∗^ for TALK, r_*part*_ = 0.28^∗∗∗^ for AVATA, r_*part*_ = 0.08^∗∗∗^ for CURIO. ^+^p < 0.10, ^*^p < 0.05, ^∗∗^p < 0.01, and ^∗∗∗^p < 0.001.

### Video Game Genre and Psychological Functioning

Third, we examined whether players’ preferences for different video game genres were differentially associated with the measures of psychological functioning. Table 3 shows the partial correlations in which we controlled for sex and age. There was a weak connection between general psychological symptoms and all of the video game genres we investigated except strategy. A preference for action games had the strongest association with affect while playing. Thus, action games seem to be both rewarding and a source of frustration. A preference for action games went along with poorer school performance. Gamers who preferred role-playing games scored higher on shyness and a preference for solitude and lower on self-esteem; they also reported fewer offline connections. By contrast, preferences for games of the unclassified category on average went along with a larger number of offline friends and more positive affect, both while playing and in general. Two game genres (i.e., role-playing and unclassified games) were related to the coping strategy of self-distraction. Because preferred game genre was related to participants’ sex (see Supplementary Table S3), we had a more detailed look at the correlations between preferred game genre and psychological functioning separately for both sexes: For males (*n* = 2,377), the strongest correlation between general psychopathology and game genre emerged for action (*r* = 0.08, *p* < 0.001), followed by role playing (*r* = 0.07, *p* < 0.01), and unclassified (*r* = 0.07, *p* < 0.01). For females (*n* = 357), the strongest relation between general psychopathology and game genre emerged for simulation (*r* = 0.17, *p* < 0.01). Differences were also found regarding the strength of the relation between number of friends online and the genre action: *r* = 0.06, *p* < 0.01 for males, and *r* = 0.27, *p* < 0.001 for females. Similarly, preferred game genre was related to participants’ age (see Supplementary Table S3). However, there were merely differences with regard to the relation of psychological functioning and game genre, when analyzed separately for different age groups (<19 years, *n* = 557; 19–30 years, *n* = 1916; >31 years, *n* = 261). In sum, our results speak to the idea that individuals with different levels of psychological functioning differ in their choices of game genres and vice versa.

**TABLE 3:** Associations between preferred video game genre and psychological functioning.

	**Video game genre**				
	***r*_part_**				
	**Simulation**	**Strategy**	**Action**	**Role-playing**	**Unclassified**
General psychopathology	0.06^∗∗^	0.01	0.06^∗∗∗^	0.07^∗∗∗^	0.07^∗∗∗^
**Coping**					
Self-distraction	0.07^∗∗^	0.04^*^	0.07^∗∗∗^	0.12^∗∗∗^	0.12^∗∗∗^
Denial	0.06^∗∗^	–0.03	0.06^∗∗^	–0.01	0.08^∗∗∗^
Substance use	–0.00	−0.04^*^	0.05^*^	0.00	0.02
Venting	0.06^∗∗^	−0.05^*^	0.04^*^	0.04^+^	0.07^∗∗∗^
Self-blame	0.02	0.03^+^	–0.01	0.06^∗∗^	0.02
Behavioral disengagement	0.05^*^	0.03	0.03^+^	0.05^*^	0.02
Acceptance	0.01	–0.01	0.06^∗∗^	0.02	0.03^+^
Active coping	–0.01	0.05^*^	0.02	0.01	0.06^∗∗^
Planning	0.03	0.09^∗∗∗^	0.03	0.02	0.05^∗∗^
Positive reframing	0.01	0.03	0.05^∗∗^	–0.00	0.09^∗∗∗^
**Affect**					
Positive affect in general	0.04^+^	0.00	0.07^∗∗∗^	–0.07^∗∗∗^	0.10^∗∗∗^
Negative affect in general	0.06^∗∗^	0.02	0.05^*^	0.09^∗∗∗^	0.04^+^
Positive affect while playing	0.01	0.07^∗∗∗^	0.21^∗∗∗^	0.06^∗∗^	0.11^∗∗∗^
Negative affect while playing	0.02	0.01	0.13^∗∗∗^	0.01	0.07^∗∗∗^
Shyness	0.03	0.04^*^	–0.02	0.13^∗∗∗^	–0.03
Loneliness	0.04^+^	0.00	–0.01	0.07^∗∗^	0.01
Preference for solitude	–0.00	0.03^+^	0.03	0.12^∗∗∗^	−0.03^+^
Life satisfaction	–0.00	–0.01	–0.01	–0.06^∗∗^	0.01
Self-esteem	–0.01	0.00	−0.03^+^	–0.12^∗∗∗^	–0.00
Self-efficacy	–0.02	0.03	0.03	–0.05^∗∗^	0.04^+^
Social support	–0.00	–0.03	–0.00	–0.05^∗∗^	0.03
Friends and acquaintances offline	0.00	–0.01	0.01	–0.10^∗∗∗^	0.09^∗∗∗^
Friends and acquaintances online	–0.01	0.07^∗∗^	0.08^∗∗∗^	0.05^∗∗^	0.05^∗∗^
Grade point average	0.06^∗∗^	–0.06^∗∗^	0.12^∗∗∗^	0.03	0.03

Ns ranged from 2,732 to 2,733. Preferred video game genres refer to Apperley’s (2006) classification and were assessed by ratings of how often various subgenres were played ranging from 1 (never) to 4 (very often). Simulation included: life simulations (e.g., The Sims), economy simulations (e.g., Sim City), sport simulations (e.g., Fifa), other simulations (e.g., Flight Simulator). Strategy included: real-time strategy games (e.g., Age of Empires), round-based strategy games (e.g., Civilization). Action included: first-person shooter single-player (e.g., Half Life), first-person shooter multiplayer (e.g., Counterstrike), third-person games (e.g., Tomb Raider). Role-playing included: MMORPGs (e.g., World of Warcraft), single-player role-playing games (e.g., Dragon Age), adventures (e.g., Monkey Island). Unclassified included: jump’n’runs (e.g., Donkey Kong), social-network applications (e.g., Farmville), browser games (e.g., Sea Fight), games of skill (e.g., Tetris), music games (e.g., Guitar Hero), brain jogging (e.g., Dr. Kawashima), online gambling and card games (e.g., Poker), and single-player card games (e.g., Solitaire). Grade point average: higher values reflect poorer school performance. Partial correlations (r_*part*_, controlled for sex and age) are presented. Partial correlations (controlled for sex and age) with the AICA-S: r_*part*_ = −0.03^+^ for simulation, r_*part*_ = 0.06^∗∗^ for strategy, r_*part*_ = 0.12^∗∗∗^ for action, r_*part*_ = 0.15^∗∗∗^ for rpg, and r_*part*_ = 0.01 for unclassified. ^+^p < 0.10, ^*^p < 0.05, ^∗∗^p < 0.01, and ^∗∗∗^p < 0.001.

### Predicting Potentially Problematic Video Game Use by Psychological Functioning Variables

In a final step, we entered all of the investigated psychological functioning variables as well as sex and age as predictors of the potentially problematic use of video games. By employing this procedure, we were able to determine the unique contribution of each psychological functioning variable when the influence of all other variables was held constant. As Table 4 shows, the number of online friends and acquaintances as well as positive affect while playing were most predictive of potentially problematic video game use over and above all other variables. General psychopathology, a lack of offline connections, and poor school performance were weaker but still relevant predictors of potentially problematic video game use.

**TABLE 4:** Prediction of potentially problematic video game use by psychological functioning variables.

		**Beta**	***p***
1	Constant		< 0.001
	Sex	0.16^∗∗∗^	< 0.001
	Age	–0.14^∗∗∗^	< 0.001
2	Constant		< 0.01
	Sex	0.11^∗∗∗^	< 0.001
	Age	–0.10^∗∗∗^	< 0.001
	General psychopathology	0.15^∗∗∗^	< 0.001
	**Coping**		
	Self-distraction	0.01	0.55
	Denial	0.03	0.11
	Substance use	0.04^*^	0.02
	Venting	−0.04^*^	0.04
	Self-blame	0.06^∗∗^	< 0.01
	Behavioral disengagement	0.01	0.56
	Acceptance	0.06^∗∗^	< 0.01
	Active coping	–0.02	0.47
	Planning	–0.07^∗∗^	< 0.01
	Positive reframing	0.00	0.98
	**Affect**		
	Positive affect in general	–0.08^∗∗∗^	< 0.001
	Negative affect in general	–0.04	0.11
	Positive affect while playing	0.20^∗∗∗^	< 0.001
	Negative affect while playing	0.09^∗∗∗^	< 0.001
	Shyness	0.01	0.75
	Loneliness	–0.02	0.27
	Preference for solitude	0.08^∗∗∗^	< 0.001
	Life satisfaction	–0.01	0.68
	Self-esteem	−0.06^*^	0.03
	Self-efficacy	0.04	0.12
	Social support	–0.01	0.71
	Friends and acquaintances offline	–0.12^∗∗∗^	< 0.001
	Friends and acquaintances online	0.21^∗∗∗^	< 0.001
	Grade point average	0.11^∗∗∗^	< 0.001

**N = 2,732. R = 0.56, R^2^ = 0.30. Grade point average: higher values reflect poorer school performance.**

## Discussion

With this study, we aimed to shed light on the association of diverse video gaming habits with gamers’ psychological functioning. Drawing on a large sample, our results revealed a medium-sized relation between potentially problematic video game use and poor psychological functioning with regard to general psychological symptoms, maladaptive coping strategies, negative affectivity, low self-esteem, and a preference for solitude as well as poor school performance. These findings are in line with those of prior work (e.g., Kuss and Griffiths, 2012; Milani et al., 2018). Also, reasons for playing video games were differentially related to psychological functioning with the most pronounced findings for escape-oriented in contrast to gain-oriented motives. Specifically, distraction-motivated gaming went along with higher symptom ratings, lower self-esteem, and more negative affectivity, whereas playing to establish social relationships in the virtual world was related to a larger number of online connections and more positive affect while playing. Furthermore, there were only weak relations between the preferred game genres and psychological functioning. The action games genre was associated with the strongest ratings of affect while playing. These results on reasons and genres may help to explain conflicting findings of former studies, because in our work we examined various reasons for playing, several game genres, and various aspects of psychological functioning simultaneously. Finally, positive affect while playing and a larger number of online friends were the strongest unique predictors of potentially problematic video game use, followed by psychological symptoms, a lack of offline connections, and poor school performance. These findings suggest that, on the one hand, independent of one’s psychological conditions, enjoying oneself during gaming (i.e., experiencing positive affect, connecting with online friends) may go along with potentially problematic use of video games. On the other hand, poor psychological functioning seems to be a unique risk factor for potentially problematic video gaming.

The presented results are generally in line with previous work that has identified a connection between video gaming and psychological health, academic problems, and social problems (Ferguson et al., 2011; Müller et al., 2015). However, our study moved beyond prior research by providing in-depth analyses of both video gaming habits (including potentially problematic use, reasons for playing, and preferred game genre) and psychological functioning (including psychological symptoms, coping styles, affectivity, as well as variables that are related to individuals and their social environments). In addition, we identified unique predictors of potentially problematic video game use.

How can the findings on differential relations between video gaming and various indices of psychological functioning – ranging from beneficial results (Latham et al., 2013) to unfavorable results (Barlett et al., 2009; Möller and Krahé, 2009; Anderson et al., 2010) – be integrated? According to Kanfer and Phillips (1970), problematic behavior (e.g., excessive video gaming) can be understood as a function of the situation (e.g., being rejected by a peer); the organism (e.g., low self-esteem); the person’s thoughts, physical reactions, and feelings (e.g., sadness, anger); and finally, the short- as well as long-term consequences of the behavior (termed SORKC model). In the short run, according to our results, playing video games may be a way to distract oneself from everyday hassles and may lead to positive affect while playing and a feeling of being connected to like-minded people, all of which are factors that have an immediate reinforcing value. In the long run, however, spending many hours per day in front of a computer screen may prevent a person from (a) developing and practicing functional coping strategies, (b) finding friends and support in the social environment, and (c) showing proper school achievement, factors that are potentially harmful to the person. Thus, differentiating between short- and long-term perspectives may help us understanding the differential correlates of intensive video gaming.

When is it appropriate to speak of video game addiction? More and more researchers have suggested a continuum between engagement (Charlton and Danforth, 2007; Skoric et al., 2009) and pathological gaming/addiction, instead of a categorical perspective. In part, this recommendation has also been followed in the DSM-5 (American Psychiatric Association, 2013) where Internet Gaming Disorder is classified with different degrees of severity, ranging from mild to moderate to severe, according to the functional impairment associated with it. The AICA-S also allows for a differential perspective on gaming behavior by providing ways to assess both the time spent playing video games and the main DSM criteria that indicate Internet Gaming Disorder. However, in our study we did not aim at making a diagnosis, but at having a closer look at potentially problematic gaming behavior and its correlates in a non-clinical sample.

In sum, it seems relevant to assess not only the extent of video game use but also the reasons behind this behavior (e.g., distraction) and the concrete rewards that come from playing (e.g., the experience of strong affect while playing action games) to fully understand the relation between video gaming and psychological functioning.

### Limitations and Future Directions

With the present study, we aimed to uncover the association between video gaming and psychological functioning. Our approach was cross-sectional and warrants interpretative caution because correlations cannot determine the direction of causation. It remains unclear whether potentially problematic gaming is a factor that contributes to the development of psychological dysfunction or whether psychological dysfunction contributes to potentially problematic gaming. Also, a third factor (e.g., preexisting mental difficulties) may produce both psychological dysfunction and potentially problematic gaming. Thus, longitudinal studies that are designed to identify the causal pathway may provide a promising avenue for future research. Future studies may also answer the question whether the link between video gaming and psychological functioning is moderated by sex, age, the reasons for playing, or the preferred game genre. In addition, it is important not to forget that the present results are based on a self-selected sample in which potentially problematic video gamers were overrepresented (e.g., Festl et al., 2013, for a representative sample). Thus, future research should replicate our findings in a representative sample. Further, we relied on self-reported data, which is a plausible method for assessing inner affairs such as people’s reasons for their behaviors, but it would be helpful to back up our findings with evidence derived from sources such as peers, caregivers, and health specialists. Our work reflects only a first approach to the topic, and future work may additionally collect in-game behavioral data from the players (McCreery et al., 2012; Billieux et al., 2013) to objectively and more specifically investigate diverse patterns of use. Furthermore, one must not forget that the used taxonomy to classify video game genres is only one of various possible options and one should “think of each individual game as belonging to several genres at once” (Apperley, 2006, p. 19). Finally, some of the effects reported in our paper were rather modest in size. This is not surprising considering the complexity and multiple determinants of human behavior. In our analyses, we thoroughly controlled for the influence of sex and age and still found evidence that video gaming was differentially related to measures of psychological functioning.

## Conclusion

The current study adds to the knowledge on gaming by uncovering the specific relations between video gaming and distinct measures of psychological functioning. Potentially problematic video gaming was found to be associated with positive affect and social relationships while playing but also with psychological symptoms, maladaptive coping strategies, negative affectivity, low self-esteem, a preference for solitude, and poor school performance. Including gamers’ reasons for playing video games and their preferred game genres helped deepen the understanding of the specific and differential associations between video gaming and psychological health. This knowledge might help developing adequate interventions that are applied prior to the occurrence of psychological impairments that may go along with potentially problematic video gaming.

## Ethics Statement

In our online survey, participants were given information on voluntary participation, risks, confidentiality/anonymity, and right to withdraw. Whilst participants were not signing a separate consent form, consent was obtained by virtue of completion. We implemented agreed procedures to maintain the confidentiality of participant data.

## Author Contributions

BB, BE, JH, and KM conceived and designed the study. BB, JH, and KM collected and prepared the data. JH analyzed the data. BE and JH wrote the manuscript.

## Conflict of Interest Statement

The authors declare that the research was conducted in the absence of any commercial or financial relationships that could be construed as a potential conflict of interest.

## Footnotes

1 The data were gathered as part of a larger project (Stopfer et al., 2015; Braun et al., 2016). However, the analyses in the present article do not overlap with analyses from previous work.

2 Other measures were administered, but they were not relevant to the present research questions and are thus not mentioned in this paper. The data set and analysis script supporting the conclusions of this manuscript can be retrieved from https://osf.io/emrpw/?view_only=856491775efe4f99b407e258c2f2fa8d.
