# Supplementary material for: The Association Between Video Gaming and Psychological Functioning
Source: Front Psychol. 2019 Jul 26;10:1731. doi: 10.3389/fpsyg.2019.01731 (PMC6676913; doi:10.3389/fpsyg.2019.01731)
Supplement: Supplementary file 1 [file Data_Sheet_1.docx]

**Supplemental online material**

**Table S1. Descriptive statistics for measures of psychological functioning.**

| **Psychological functioning** |  | **Min** | **Max** | ***M*** | ***SD*** |  | **Sex** | **Age** |
| --- | --- | --- | --- | --- | --- | --- | --- | --- |
| General psychopathology |  | 1 | 5 | 1.89 | .64 |  | -.15*** | -.11*** |
| Coping |  |  |  |  |  |  |  |  |
| Self-distraction |  | 1 | 5 | 2.89 | .80 |  | -.12*** | -.09*** |
| Denial |  | 1 | 5 | 1.68 | .80 |  | -.01 | -.09*** |
| Substance use |  | 1 | 5 | 1.57 | .95 |  | .05* | .05* |
| Venting |  | 1 | 5 | 1.88 | .94 |  | -.27*** | -.04* |
| Self-blame |  | 1 | 5 | 2.78 | 1.06 |  | -.07*** | -.08*** |
| Behavioral disengagement |  | 1 | 5 | 1.70 | .80 |  | -.02 | -.06** |
| Acceptance |  | 1 | 5 | 2.77 | 1.06 |  | .02 | -.06** |
| Active coping |  | 1 | 5 | 3.45 | .81 |  | -.08*** | .08*** |
| Planning |  | 1 | 5 | 3.60 | .91 |  | -.09*** | .10*** |
| Positive reframing |  | 1 | 5 | 3.31 | .86 |  | -.03 | .05** |
| Affect |  |  |  |  |  |  |  |  |
| Positive affect in general |  | 1.3 | 5 | 3.41 | .55 |  | .03 | -.02 |
| Negative affect in general |  | 1 | 5 | 1.87 | .60 |  | -.07*** | -.06*** |
| Positive affect while playing |  | 1 | 5 | 3.48 | .68 |  | .14*** | -.05** |
| Negative affect while playing |  | 1 | 5 | 1.56 | .50 |  | .10*** | -.12*** |
| Shyness |  | 1 | 5 | 2.62 | .95 |  | -.04+ | -.05** |
| Loneliness |  | 1 | 5.25 | 2.41 | .76 |  | -.08*** | -.12*** |
| Preference for solitude |  | 1 | 6 | 3.77 | .97 |  | -.09*** | .11*** |
| Life satisfaction |  | 1 | 4 | 3.00 | .85 |  | .03 | -.03 |
| Self-esteem |  | 1.10 | 4 | 3.24 | .56 |  | .06** | .11*** |
| Self-efficacy |  | 1 | 4 | 2.99 | .46 |  | .09*** | .06** |
| Social support |  | 1 | 5 | 4.28 | .82 |  | -.08*** | .08*** |
| Friends offline |  | 0 | 120 | 5.42 | 5.12 |  | .09*** | -.11*** |
| Acquaintances offline |  | 0 | 900 | 57.78 | 78.81 |  | .09*** | -.04* |
| Friends online |  | 0 | 60 | .47 | 1.64 |  | .02 | -.01 |
| Acquaintances online |  | 0 | 520 | 8.38 | 24.99 |  | .10*** | -.04* |
| Grade point average |  | 1 | 6 | 2.43 | .60 |  | .17*** | -.05** |

*Note.* *Ns* ranged from 2,712 to 2,734. Due to left-skewed distributions, the number of offline friends and the number of offline acquaintances as well as the number of online friends and the number of online acquaintances were logarithmized before aggregation. Here, statistics for the original (nonlogarithmized) data are presented. Grade point average: higher values reflect poorer school performance. Sex was coded 1 = female and 2 = male. * *p* < .05. ** *p* < .01. *** *p* < .001.

**Table S2. Descriptive statistics for participants’ reasons for playing video games.**

|  | |  |  |  |  |  |  | **Correlations with** | | | | | | | | | | |
| --- | --- | --- | --- | --- | --- | --- | --- | --- | --- | --- | --- | --- | --- | --- | --- | --- | --- | --- |
| **Reasons for playing** | |  | **Min** | **Max** | ***M*** | ***SD*** |  | **2** | **3** | **4** | **5** | **6** | **7** | **8** | **9** | **10** | **Sex** | **Age** |
| 1 | Amusement |  | 1 | 4 | 2.94 | .85 |  | .31*** | .10*** | .15*** | .03 | .02 | .10*** | .09*** | .11*** | .11*** | -.00 | -.07** |
| 2 | Distraction |  | 1 | 4 | 2.35 | .92 |  |  | .05** | .25*** | .11*** | .13*** | .11*** | .15*** | .13*** | .11*** | .02 | -.04* |
| 3 | Storyline |  | 1 | 4 | 2.67 | 1.10 |  |  |  | .27*** | .44*** | .11*** | -.08** | .11*** | .23*** | .39*** | .03 | .08*** |
| 4 | Relaxation |  | 1 | 4 | 2.96 | .91 |  |  |  |  | .30*** | .13*** | .06** | .11*** | .17*** | .22*** | -.00 | .06** |
| 5 | Imagination |  | 1 | 4 | 1.93 | 1.04 |  |  |  |  |  | .32*** | .07*** | .18*** | .24*** | .34*** | -.07*** | .04* |
| 6 | Real-life abilities |  | 1 | 4 | 1.38 | .70 |  |  |  |  |  |  | .26** | .28*** | .17*** | .15*** | .05** | -.06** |
| 7 | Social relations |  | 1 | 4 | 2.02 | 1.06 |  |  |  |  |  |  |  | .28*** | .18*** | -.02 | .11*** | -.06** |
| 8 | Talk about |  | 1 | 4 | 1.34 | .65 |  |  |  |  |  |  |  |  | .23*** | .22*** | .09*** | -.07*** |
| 9 | Avatar’s abilities |  | 1 | 4 | 1.89 | .99 |  |  |  |  |  |  |  |  |  | .32*** | -.03 | .08*** |
| 10 | Curiosity |  | 1 | 4 | 2.31 | .95 |  |  |  |  |  |  |  |  |  |  | -.03 | .06** |

*Note.* *Ns* ranged from 2,712 (curiosity) to 2,727 (storyline). Reasons for playing were assessed on a Likert scale ranging from 1 (*never*) to 4 (*very often*). Sex was coded 1 = female and 2 = male. * *p* < .05. ** *p* < .01. *** *p* < .001.

**Table S3. Descriptive statistics for preferred video game genres.**

|  |  |  |  |  |  |  |  | **Correlations with** | | | | | |
| --- | --- | --- | --- | --- | --- | --- | --- | --- | --- | --- | --- | --- | --- |
| **Video game genre** |  | **α** | **Min** | **Max** | ***M*** | ***SD*** |  | **Strategy** | **Action** | **Role-playing** | **Unclassified** | **Sex** | **Age** |
| Simulation |  | .38 | 1 | 3.75 | 1.58 | .44 |  | .20*** | .16*** | .08*** | .24*** | .01 | -.03 |
| Strategy |  | .51 | 1 | 4.00 | 2.13 | .80 |  |  | .11*** | .29*** | .07*** | .22*** | .00 |
| Action |  | .63 | 1 | 4.00 | 2.54 | .84 |  |  |  | .13*** | .12*** | .39*** | -.20*** |
| Role-playing |  | .39 | 1 | 4.00 | 2.01 | .73 |  |  |  |  | .19*** | .01 | .14*** |
| Unclassified |  | .59 | 1 | 3.50 | 1.54 | .39 |  |  |  |  |  | -.20*** | .02 |

*Note.* *N* = 2,733. Preferred video game genres refer to Apperley’s (2006) classification and were assessed by ratings of how often various subgenres were played, ranging from 1 (*never*) to 4 (*very often*). Simulation included: life simulations (e.g., The Sims), economy simulations (e.g., Sim City), sport simulations (e.g., Fifa), other simulations (e.g., Flight Simulator). Strategy included: real-time strategy games (e.g., Age of Empires), round-based strategy games (e.g., Civilization). Action included: first-person shooter single-player (e.g., Half Life), first-person shooter multiplayer (e.g., Counterstrike), third-person games (e.g., Tomb Raider). Role-playing included: MMORPGs (e.g., World of Warcraft), single-player role-playing games (e.g., Dragon Age), adventures (e.g., Monkey Island). Unclassified included: jump’n’runs (e.g., Donkey Kong), social-network applications (e.g., Farmville), browser games (e.g., Sea Fight), games of skill (e.g., Tetris), music games (e.g., Guitar Hero), brain jogging (e.g., Dr. Kawashima), online gambling and card games (e.g., Poker), and single-player card games (e.g., Solitaire). Sex was coded 1 = female and 2 = male. *** *p* < .001.

**Table S4. Correlations between reasons for playing video games and preferred video game genres.**

|  | |  | **Video game genre** | | | | |
| --- | --- | --- | --- | --- | --- | --- | --- |
| **Reasons for playing** | |  | **Simulation** | **Strategy** | **Action** | **Role-playing** | **Unclassified** |
| 1 | Amusement |  | .05** | .09*** | .12*** | .11*** | .08*** |
| 2 | Distraction |  | .05** | .05* | .12*** | .09*** | .07*** |
| 3 | Storyline |  | .12*** | .16*** | .28*** | .51*** | .15*** |
| 4 | Relaxation |  | .07*** | .09*** | .15*** | .18*** | .08*** |
| 5 | Imagination |  | .11*** | .09*** | .13*** | .31*** | .14*** |
| 6 | Real-life abilities |  | .10*** | .06** | .13*** | .10*** | .13*** |
| 7 | Social relations |  | -.04* | .09*** | .13*** | .06** | -.01 |
| 8 | Talk about |  | .09*** | .05** | .24*** | .07*** | .14*** |
| 9 | Avatar’s abilities |  | .05* | .05* | .12*** | .35*** | .11*** |
| 10 | Curiosity |  | .15*** | .14*** | .19*** | .30*** | .19*** |

*Note.* *Ns* ranged from 2,712 (curiosity) to 2,727 (storyline). Reasons for playing were assessed on a Likert scale ranging from 1 (*never*) to 4 (*very often*). Preferred video game genres refer to Apperley’s (2006) classification and were assessed by ratings of how often various subgenres were played, ranging from 1 (*never*) to 4 (*very often*). Simulation included: life simulations (e.g., The Sims), economy simulations (e.g., Sim City), sport simulations (e.g., Fifa), other simulations (e.g., Flight Simulator). Strategy included: real-time strategy games (e.g., Age of Empires), round-based strategy games (e.g., Civilization). Action included: first-person shooter single-player (e.g., Half Life), first-person shooter multiplayer (e.g., Counterstrike), third-person games (e.g., Tomb Raider). Role-playing included: MMORPGs (e.g., World of Warcraft), single-player role-playing games (e.g., Dragon Age), adventures (e.g., Monkey Island). Unclassified included: jump’n’runs (e.g., Donkey Kong), social-network applications (e.g., Farmville), browser games (e.g., Sea Fight), games of skill (e.g., Tetris), music games (e.g., Guitar Hero), brain jogging (e.g., Dr. Kawashima), online gambling and card games (e.g., Poker), and single-player card games (e.g., Solitaire). * *p* < .05. ** *p* < .01. *** *p* < .001.
